# Novel *FKS1* and *FKS2* modifications in a high-level echinocandin resistant clinical isolate of *Candida glabrata*

**DOI:** 10.1080/22221751.2019.1684209

**Published:** 2019-11-12

**Authors:** Xin Hou, Kelley R. Healey, Erika Shor, Milena Kordalewska, Cristina Jiménez Ortigosa, Padmaja Paderu, Meng Xiao, He Wang, Ying Zhao, Li-Yan Lin, Yan-Hai Zhang, Yong-Zhe Li, Ying-Chun Xu, David S. Perlin, Yanan Zhao

**Affiliations:** a Department of Clinical Laboratory, Peking Union Medical College Hospital, Chinese Academy of Medical Sciences, Beijing, People’s Republic of China; b Graduate School, Peking Union Medical College, Chinese Academy of Medical Sciences, Beijing, People’s Republic of China; c Beijing Key Laboratory for Mechanisms Research and Precision Diagnosis of Invasive Fungal Diseases (BZ0447), Beijing, People’s Republic of China; d Center for Discovery and Innovation, Hackensack Meridian Health, Nutley, NJ, USA; e Department of Biology, William Paterson University, Wayne, NJ, USA; f School of Medicine, Peking University Health Science Center, Beijing, People’s Republic of China; g Central Laboratory, Hebei Yanda Hospital, Langfang, People’s Republic of China; h Department of Medical Sciences, Hackensack Meridian School of Medicine at Seton Hall University, Nutley, NJ, USA

**Keywords:** *Candida glabrata*, *FKS*, echinocandin resistance, CRISPR-Cas9, resistance mechanism

## Abstract

Echinocandin resistance in *Candida glabrata* poses a serious clinical challenge. The underlying resistance mechanism of a pan-echinocandin-resistant *C. glabrata* isolate (strain L74) was investigated in this study. *FKS* mutants carrying specific mutations found in L74 were reconstructed by the Alt-R CRISPR-Cas9 system (Fks1 WT/Fks2-E655K, strain CRISPR 31) and site-directed mutagenesis (strain *fks1Δ*/Fks2-E655K). Sequence analysis of strain L74 revealed a premature stop codon W508stop in *FKS1* and an E655K mutation preceding the hotspot 1 region in *FKS2*. Introduction of the Fks2-E655K mutation in ATCC 2001 (strain CRISPR 31) conferred a modest reduction in susceptibility. However, the same *FKS2* mutation in the *fks1Δ* background (strain *fks1Δ*/Fks2-E655K) resulted in high levels of resistance to echinocandins. Glucan synthase isolated from L74 was dramatically less sensitive to micafungin (MCF) relative to ATCC 2001. Both *FKS1/FKS2* transcript ratios and Fks1/Fks2 protein ratios were significantly lower in L74 and *fks1Δ*/Fks2-E655K compared to ATCC 2001 and CRISPR 31 (*P* <0.05). Mice challenged with CRISPR 31 and *fks1Δ*/Fks2-E655K mutants failed to respond to MCF. In conclusion, the high-level of echinocandin resistance in the clinical isolate of *C. glabrata* L74 was concluded to result from the combination of null function of Fks1 and the point mutation E655K in Fks2.

## Introduction

Invasive candidiasis is an important health-care-associated fungal infection caused by *Candida* spp. [1–3]. While *Candida albicans* is the most frequently reported *Candida* species*,* non-*albicans Candida* have an increasing role, particularly in high-risk populations, with *Candida glabrata* being one of the most prominent species [1,3,4]. *C. glabrata* is of particular concern due to its high rate of azole resistance [5]. Moreover, echinocandin resistance in *C. glabrata* has increased and posed a serious clinical challenge [6,7]. Most epidemiological prevalence studies report echinocandin resistance of 2–4% in *C. glabrata* [7]. However, some institutional studies have reported higher rates, close to or over 10% [6]. In China, *C. glabrata* echinocandin resistance is <1% [8], and we previously reported the first case of invasive candidiasis in China due to a pan-echinocandin-resistant *C. glabrata* isolate. This clinical isolate showed very high MICs (≥ 8 µg/ml) to all three echinocandins: anidulafungin (ANF), caspofungin (CSF) and micafungin (MCF) [9].

Clinical echinocandin resistance in *C. glabrata* is generally associated with amino acid substitutions in specific hotspot (HS) regions of *FKS1* and *FKS2*, which encode the drug target β-1, 3-D-glucan synthase (GS) [10]. However, partial *FKS* sequence analysis found that the high-level echinocandin resistant *C. glabrata* isolate mentioned above had no *FKS* HS mutations, but carried a single nucleotide mutation leading to a predicted E655 K alteration (3 amino acids upstream of the start of *FKS2* HS1). In this study, we performed comprehensive analyses, both *in vitro* and *in vivo,* to assess the role of this novel mutation in the high-level echinocandin resistance observed in this particular clinical isolate of *C. glabrata*.

## Materials and methods

### Ethics statement

The study was approved by the Human Research Ethics Committee of Peking Union Medical College Hospital (No. S-263). In this study, involving human subjects, informed consent has been waived and approved by the institutional review board. Animal experiments were performed at the Public Health Research Institute’s Animal Biosafety Level-2 Research Animal Facility (ICPH RAF), a centre of the New Jersey Medical School, Rutgers University (NJMS-Rutgers). Our animal facility follows the Public Health Service and National Institute of Health Policy of Humane Care and Use of Laboratory Animals. All experimental protocols were approved by the Rutgers Institutional Animal Care and Use Committee (IACUC).

### Strains, media, and drugs

*C. glabrata* strain ATCC 2001 and ATCC 200989 (2001 HTU) [11] were obtained from the American Type Culture Collection (Manassas, VA). The clinical isolate L74 was obtained as described in previous report [9], and the *fks1Δ* disruptant was a gift from Dr. Santosh Katiyar (Drexel University College of Medicine) [12]. The medium was 1% yeast extract, 2% peptone, and 2% dextrose (YPD). ANF was obtained from Pfizer (New York, NY), CSF was obtained from Merck (Rahway, NJ), MCF was obtained from Astellas Pharma (Deerfield, IL), and FK506 was obtained from Tecoland (Edison, NJ). PCR primers were purchased from Integrated DNA Technologies (Coralville, IA).

### *FKS* genes sequencing

The entire DNA coding sequences of *FKS1* (5592 bp) and *FKS2* (5694 bp) were amplified and determined, using primers listed in Supplementary Table 1. Amplicons were purified by using a ZR DNA sequencing cleanup kit (Zymo Research, Irvine, CA, USA) and sequenced by Genewiz (South Plainfield, NJ, USA). Sequencing results were analyzed by SeqMan Pro (version 14) software (Lasergene, DNAStar). The nucleotide sequences of *FKS1* and *FKS2* were aligned and compared with the reference wild-type (WT) sequences of *C. glabrata* ATCC 90030 *FKS1* (GenBank accession numbers: HM366440.1) and *FKS2* (GenBank accession numbers: HM366442.1), respectively.

### Antifungal susceptibility tests

Antifungal susceptibility testing was performed according to CLSI guidelines [13] and in complete synthetically defined (SD) medium (Sunrise Science Products) to compensate for auxotrophies, as described previously [12]. Serial two-fold dilutions of FK506 ranging from 0.25 to 64 µg/ml were added to cultures prior to aliquoting to the 96-well plates to test the susceptibility.

### Construction of C. glabrata fks2 mutants

*C. glabrata* strain ATCC 2001 with a single mutation E655 K in Fks2 was constructed by the Alt-R CRISPR-Cas9 system (Integrated DNA Technologies, Inc.) [14]. All oligonucleotides used are listed in Table S1. Transformations were performed by electroporation of competent cells prepared using lithium acetate (LiAc) [15]. Electroporation was performed using an 0.2 cm electroporation cuvette and electroporated with a manual 1.8 pulse (Bio-Rad MicroPulser), as described previously [14]. Another E655 K mutant in the *fks1Δ* background was constructed by transforming with a PCR-amplified *FKS2* fragment harbouring Fks2-E655K point mutation. Transformants were selected on 0.5 µg/ml CSF YPD plate and validated by PCR for correct insertion. *FKS1* and *FKS2* were subsequently sequenced to ensure the absence of any off-target mutations.

### Measurement of inhibition of glucan synthase by MCF

All isolates used in this work were grown with vigorous shaking at 37°C to early stationary phase in YPD broth, and cells were collected by centrifugation. Cell disruption, membrane protein extraction, and partial GS purification by-product entrapment were performed, as described previously [16]. Sensitivity to MCF was measured in a polymerization assay, using a 96-well multiscreen high-throughput screen filtration system (Millipore corporation, Bedford, MA) with a final volume of 100 µl [16]. Serial dilutions of MCF (0.01–10,000 ng/ml) were used to determine 50% inhibitory concentration (IC_50_) values. The reactions were initiated by addition of the purified GS. Inhibition profiles and IC_50_ values were determined using a sigmoidal response (variable-slope) curve-fitting algorithm with GraphPad Prism 6.05 software (Prism Software, Irvine, CA). Each assay was repeated three times.

### RNA extraction and quantitative real-time reverse-transcription (RT)-PCR

*C. glabrata* cells were grown in YPD broth to log phase and total RNA was extracted using the RNeasy Mini kit (QIAGEN Science, Maryland, USA) following the manufacturer’s instructions. The RNA was then treated with RNase-free DNase (Thermo Fisher Scientific, USA) according to the manufacturer’s recommendations. RNA samples were stored at −80°C. The expression levels of *FKS1* and *FKS2* were measured by RT–PCR, using One Step SYBR PrimeScript RT–PCR Kit II (TaKaRa, Shiga, Japan). Reactions were run on Mx3005P qPCR System (Agilent Technologies, CA, USA) containing 10 ng RNA sample, 0.4 μM of each primer (Table S1), 12.5 μl 2 ×  One Step SYBR RT–PCR Buffer, and 1 μl PrimeScript 1 step Enzyme Mix 2 in a volume of 25 μl. Thermal cycling conditions were as follows: RT at 42°C for 5 min; PCR cycling with initial denaturation at 95°C for 10 s, followed by 40 cycles of denaturation at 95°C for 5 s and annealing and elongation at 60°C for 20 s; a post PCR melting curve analysis with 95°C for 5 s, 60°C for 1 min then increasing to 95°C with a ramp rate of 0.5°C/s. Each experiment was carried out in triplicate and negative controls were included in each run. The RDN5.8 gene was used as reference gene to normalize the data [17]. Comparative quantitation analyses were performed using the 2^−ΔΔCT^ method [18]. The fold changes were determined from the mean normalized expression of isolates relative to the mean normalized expression of ATCC 2001 *FKS1*.

Statistical analysis of gene expression was carried out using the student’s t test by SPSS software (version 12.0, SPSS Inc., Chicago, USA), and *P* value of <0.05 was considered significant.

### Western blot

The GS was prepared with reducing buffer and Tris-Glycine SDS sample buffer (Thermo Fisher Scientific, USA), separated by electrophoresis (8% Tris-Glycine gel) (Thermo Fisher Scientific) and transferred to PVDF membrane. Fks1 and Fks2 proteins were detected with New Zealand rabbit polyclonal antibodies (GenScript, NJ, USA), which were raised against a 15 amino acid epitope in the N-terminus of each target protein and purified by SDS-PAGE, diluted 1:5000 in blocking buffer overnight at 4°C. Horseradish peroxidase-coupled donkey anti-rabbit IgG (1:3000 dilution, Cell Signaling Technology, Boston, MA, USA) was incubated for 1 h at 37°C followed by washing. The protein was revealed with Novex® ECL Chemiluminescent substrates (Thermo Fisher Scientific) per the manufacturer’s instructions. The densitometry of the blot was analyzed by Image J. Statistical analysis of gene expression was carried out using the student’s t test by SPSS software, and *P* value of <0.05 was considered significant.

### *In vivo* MCF response assessment in a murine model of invasive candidiasis

A well-established neutropenic mouse model of disseminated candidiasis was used for this study [19]. A total of 60 female eight-week-old BALB/c mice (Charles River Laboratories) were randomized into 12 different infection/antifungal therapy arms. The sample size of this animal experiment was considered adequate on the basis of the resource equation method [20]. Mice were rendered neutropenic by receiving cyclophosphamide at 150 mg/kg on day −4 and 100 mg/kg on day −1 prior to infection, and day + 2 after infection via intraperitoneal (i.p.) injection. *C. glabrata* WT ATCC 2001, CRISPR 31 and *fks1Δ*/Fks2-E655 K were used in the study. The organisms were subcultured in YPD broth at 37°C with shaking overnight. Cells were collected by centrifugation, washed twice with sterile phosphate-buffered saline (PBS), and counted with a hemocytometer. The inoculum was adjusted to 4 × 10^7^ CFU/ml, and 50 µl was used to infect each mouse. The actual infection dose was verified by determination of viable counts on YPD plates spread with proper dilutions of the inoculum and incubated at 37°C for 24 h. On day 0, mice were infected with 2 × 10^6^ CFU of the respective *C. glabrata* strain via retro-orbital injection. At 2 h post inoculation, groups of 10 mice were given single or multiple daily treatment of vehicle (PBS) or MCF at 5 mg/kg via i.p. injection. Single dosed mice were sacrificed at 24 h post inoculation, and all mice receiving multiple doses of treatment were sacrificed at day 3 post inoculation, via CO_2_ inhalation and kidneys were aseptically removed for enumeration of the fungal burdens. All graphical data were expressed as scattered data points with means and standard deviations (bars) and were statistically analyzed by analysis of variance (ANOVA) using Prism computer software. The differences in the burdens between the test and the control groups were assessed by *post hoc* analyses, using Dunnett’s or Dunn’s multiple-comparison test (when grou*p* values did not fit a Gaussian distribution). A *P* value of <0.05 was considered statistically significant.

## Results

### Characterization of the echinocandin-resistant clinical isolate

The MICs for the clinical strain L74 were 16 µg/ml for CSF, 16 µg/ml for MCF and 8 µg/ml for ANF. The DNA sequence analysis of the complete open reading frame (ORF) of *FKS1* revealed a non-synonymous mutation (G1524A), resulting in a premature stop codon (TGA; W508stop) predicted to truncate the 1863 amino acid-long Fks1 protein after the first 507 amino acids. The *FKS2* sequence analysis confirmed the pre-HS mutation G1963A (E655 K) [9].

### Antifungal susceptibilities of mutant strains

The parent strains ATCC 2001, ATCC 200989, and *fks1Δ* disruptant were susceptible to all echinocandins (Table 1). The introduction of the point mutation G1963A in *FKS2* (Fks2p E655 K; strain CRISPR 31) in WT strain ATCC 2001 using Alt-R CRISPR-Cas9 system resulted in a slight reduction of susceptibility to echinocandins (0.25 µg/ml, Table 1), 32-64-fold lower than that observed for the clinical isolate L74. However, when the same mutation was introduced in the *fks1Δ* background (strain *fks1Δ*/ Fks2-E655K), a strong resistant phenotype (MICs ≥ 8 µg/ml, Table 1) was observed, recapitulating that of the clinical isolate L74. As previously reported [12], *FKS1* disruption alone did not alter echinocandin susceptibility (Table 1). The calcineurin inhibitor FK506, which suppresses *FKS2* expression [12], did not inhibit the growth of strains ATCC 2001, ATCC 200989, and CRISPR-31, but it was highly active against strains in the *fks1Δ* or nonfunctional Fks1 protein (L74) background. The high levels of echinocandin resistance in strains *fks1Δ* /Fks2-E655 K and L74 were reversed by FK506 *in vitro* (Table 1).

**Table 1.** Echinocandin susceptibilities of C. glabrata wild-type and mutant strains, and reversal of Fks2-mediated echinocandin resistance by FK50**6**.

Strain	Predicted amino acid change		MIC (µg/ml)				
	Fks1p	Fks2p	CAS	MCF	ANF	FK506	MCF + FK506 (4 µg/ml)
ATCC 2001	WT	WT	0.25	≤0.03	0.06	32	≤0.03
ATCC 200989	WT	WT	0.25	0.12	0.06	32	≤0.03
*fks1Δ*	deleted	WT	0.25	0.12	0.06	≤0.25	≤0.03
*fks1Δ* /Fks2-E655K	deleted	E655K	8	16	8	≤0.25	≤0.03
L74	W508stop	E655K	16	16	8	≤0.25	≤0.03
CRISPR-31	WT	E655K	0.25	0.25	0.25	32	≤0.03

*CAS, caspofungin; MCF, micafungin; ANF, anidulafungin*.

### Inhibition of glucan synthase (GS) activity by MCF

**Figure 1.**
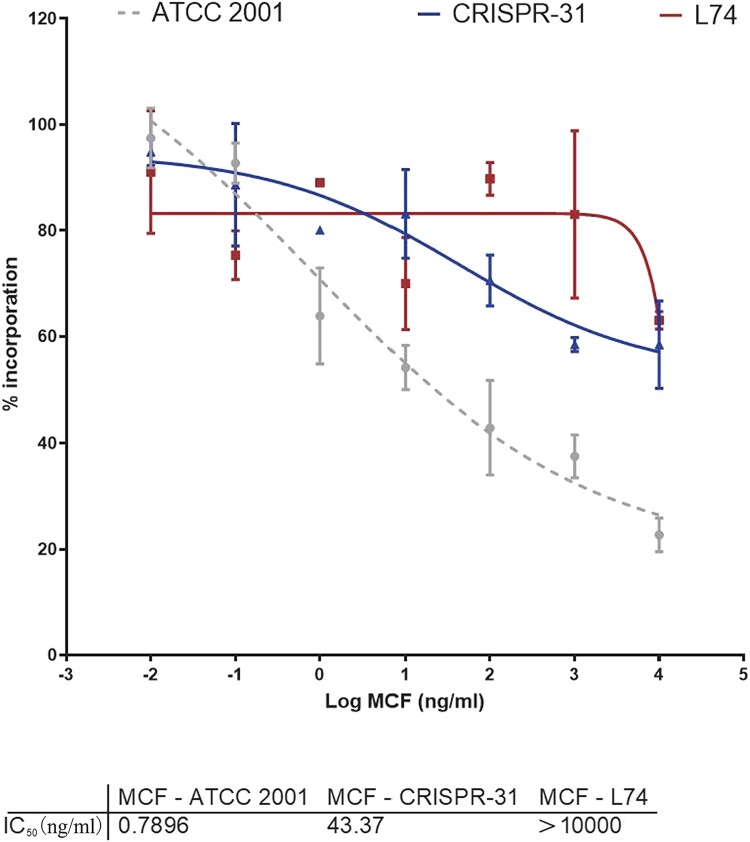
Micafungin (MCF) inhibition profiles of trapped GS complexes from wild-type ATCC 2001 (grey circles), *FKS2* E655 K mutant CRISPR 31 (blue triangles), and *FKS1* W508stop and *FKS2* E655 K clinical L74 (red squares) strains. The data are presented as the mean ± s.d.

We assessed kinetic inhibition of GS activity by MCF by determining IC_50_ values for GS enzymes partially purified from strains ATCC 2001, CRISPR 31 and L74. GS extracted from the susceptible strain ATCC 2001 was sensitive to MCF (IC_50 _= 0.7896 ng/ml). The strain CRISPR 31 had increased IC_50_ (43.37 ng/ml) relative to the WT strain. The MCF IC_50_ (> 10,000^ ^ng/ml) for the clinical resistant strain L74 harbouring *FKS1* W508stop and *FKS2* E655 K was dramatically higher than that for the WT or *FKS2* E655 K strains (Figure 1). Thus, the results of GS inhibition analysis were consistent with the high MICs.

### Expression of *FKS* and abundance of GS

**Figure 2.**
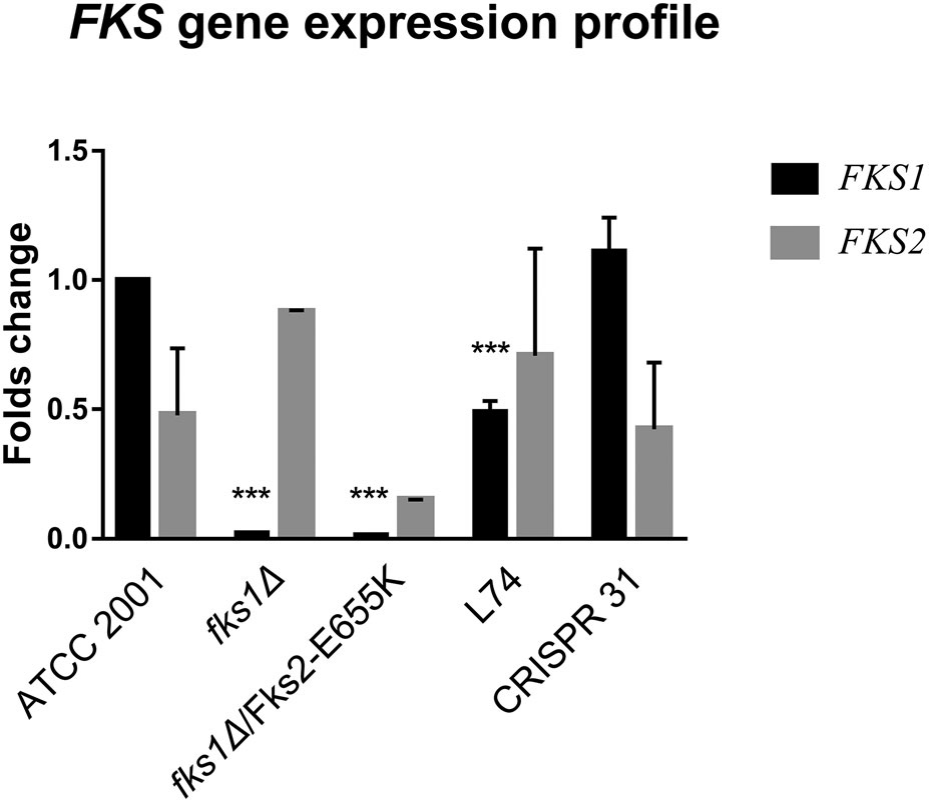
Relative expression of *FKS1* and *FKS2* genes in *C. glabrata* mutants determined by RT–PCR. Statistical analysis was performed using Student’s t-test (*** *P* < 0.01). The data are presented as the mean ± s.d.

As shown in Figure 2, at the transcript level *FKS1* and *FKS2* were expressed at a ∼ 2:1 ratio in strain ATCC 2001. Relative to ATCC 2001, *FKS1* was barely expressed in *fks1Δ* strains, and its expression was significantly decreased (*P *< 0.01) in strain L74. *FKS1* expression in CRISPR 31 was similar to that in ATCC 2001. *FKS2* expression was not significantly different among the tested strains. As a result, the *FKS1/FKS2* expression ratio was significantly lower in L74 and *fks1Δ*/ Fks2-E655 K than that in ATCC 2001 (*P *< 0.01). *FKS1/FKS2* expression ratio similar to that of ATCC 2001 was detected in CRISPR 31.

**Figure 3.**
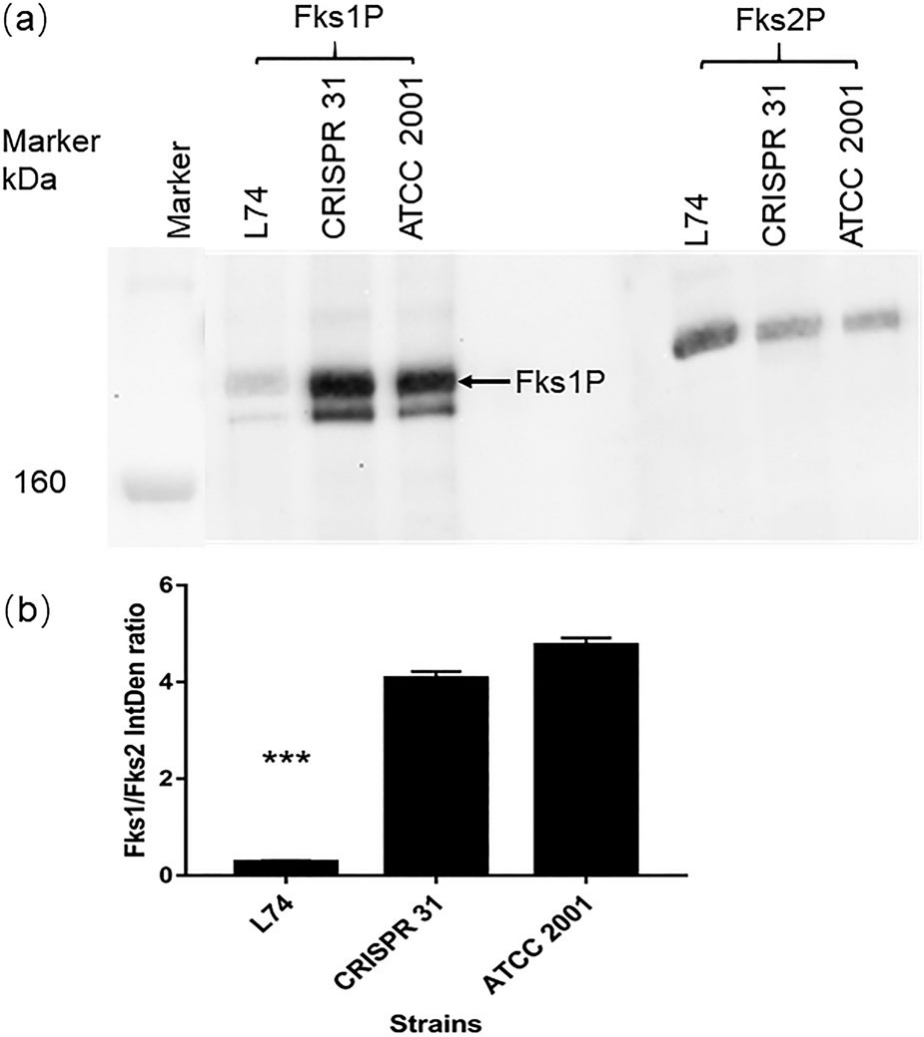
Expression of Fks1 and Fks2 in ATCC 2001, CRISPR 31 and L74. (a) Immunoblot analysis of Fks1 and Fks2. (b) The densitometry of the blot was analyzed by Image J. Statistical analysis was performed using Student’s t-test (*** *P* < 0.01). The data are presented as the mean ± s.d.

The relative protein abundance of Fks1 and Fks2 was determined for strains CRISPR 31, L74, and ATCC 2001 by Western blot analysis (Figure 3(a)). The Fks2 antibody was highly specific with a single band detected at the target molecular weight site for all strains. In the Fks1 blot, an unspecific band with lower molecular weight than Fks1 was visualized for all strains, but it was not considered to have a directional bias on the Fks1/Fks2 ratio estimation, as the unspecific hybridization products appeared universally in all tested strains and proportional to the amount of Fks1. Of note, we did not observe any visible band at lower molecular weight deemed for the truncated Fks1 protein from the L74. The Fks1 blot of L74 had very faint “WT-like” protein bands (possibly due to non-specific binding with Fks2 protein), very similar to what we saw with the *fks1Δ* strain tested in another study (data not shown). Hence, protein expression ratio calculation was based on the band densities shown at WT Fks1 and Fks2 molecular weight for comparable convenience. The Fks1/Fks2 protein ratio was > 4 for ATCC 2001 and CRISPR 31, but it was completely reversed (< 0.3) in L74 (*P *< 0.01) (Figure 3(b)).

### *In vivo* response to MCF

**Figure 4.**
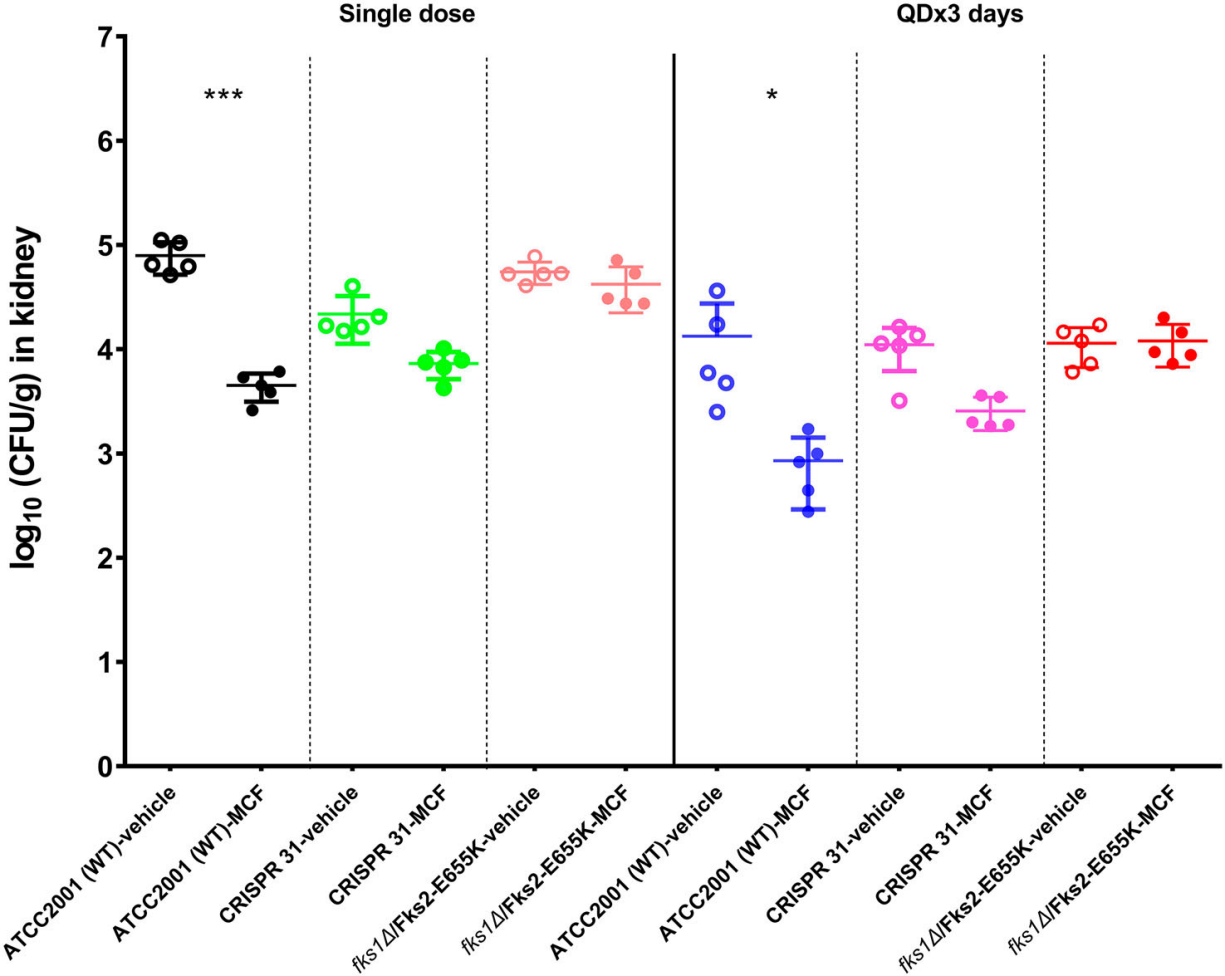
Comparison of the kidney burdens among the different treatment groups of mice infected with *C. glabrata FKS1* WT isolate (ATCC 2001), *FKS2* E655 K CRISPR 31 and *fks1Δ*/Fks2-E655 K. Statistical analysis was performed using Dunnett’s or Dunn’s multiple-comparison test (*** *P* < 0.01 and * *P* < 0.05). The data are presented as the mean ± s.d.

We compared kidney burdens of mice infected with E655 K mutants or WT strain and treated with MCF or vehicle control. After a single dose of MCF treatment, the WT strain infected mice had 1.3 log_10_ CFU/g kidney burden reduction relative to the control mice (*P* < 0.01). In comparison, the burden reduction was only 0.5 and 0.1 log_10_ CFU/g in mice infected with CRISPR 31 and *fks1Δ*/Fks2-E655 K, respectively, which was not significantly different from those infected with the same strain but treated with vehicle control (Figure 4). Similar results were observed in mice which received 3 days treatment. An average of 1.2 log_10_ CFU/g kidney burden reduction was obtained in WT strain infected mice, as a result of three doses of MCF treatment compared to vehicle control. In contrast, MCF was not effective in treating mice infected with E655 K mutants, as kidney burdens relative to those treated with vehicle control were either slightly reduced (0.6 log_10_ CFU/g, *P* > 0.05) in CRISPR 31 infected mice or not decreased at all in *fks1Δ*/Fks2-E655 K infected mice.

## Discussion

*C. glabrata* is the fourth most common *Candida* species detected in bloodstream isolates in China and the second or third in the United States, as well as in northern and eastern areas of Europe [8,21]. The propensity of *C. glabrata* to readily acquire resistance to major antifungal drug classes (azole and echinocandin) has been a concern [22]. Despite the overall prevalence of echinocandin resistance is low in China (< 1%) [8], monitoring of antifungal susceptibility trends in *C. glabrata* is warranted. In this study, we investigated the mechanism of resistance in a high-level echinocandin resistant clinical isolate of *C. glabrata* obtained from an intra-abdominal candidiasis (IAC) patient [9].

Even though *FKS1* and *FKS2* HS mutations is the major mechanism of echinocandin resistance in *C. glabrata* [10], no *FKS* mutation within the proposed HS1 was found in this particular clinical isolate. Nevertheless, an E655 K mutation just upstream of the HS1 region of *FKS2* and a premature stop codon in *FKS1* were revealed by sequence analysis of the entire ORF of both *FKS* genes. We then dissected the contribution of these two mutational events to echinocandin resistance. Introduction of the E655 K single mutation in the WT background conferred slight MIC increase, whereas the same mutation introduced into the *fks1Δ* background led to a strong echinocandin resistant phenotype very similar to that of L74. Moreover, this strong resistance could be reversed by suppression of *FKS2* expression with the calcineurin inhibitor FK506, indicating that resistance was mediated by this novel *FKS2* mutation E655 K but at the cost of functional Fks1. Our findings are in agreement with a previous study, which demonstrated that the Fks1-Fks2 redundancy attenuates the impact of resistance-conferring HS mutations [12]. Interestingly, our study is a counterpart of a previous report, in which introduction of a HS mutation in *FKS1* alone conferred an intermediate reduction in susceptibility, a premature stop codon in *FKS2* alone had no effect on susceptibility, but the combination of both severely reduced susceptibility [23].

In light of the previous finding that Fks1 and Fks2 are differentially regulated and their expression levels may impact echinocandin resistance level conferred by *FKS* mutations [12], we examined both the RNA and protein expression pattern of Fks in the clinical isolate, constructed mutants, and WT strains. The *fks1* null mutants had only minimal RNA expression of *FKS1*, as expected, and transcriptional levels of *FKS1* for L74 was also significantly decreased, while the expression level of *FKS2* increased.

Previous exposure to echinocandins has been clearly recognized as a risk factor for the development of resistance [24]. In our case, L74 was isolated from an IAC patient without any known prior antifungal exposure within 30 days of isolation [9], however, we cannot completely rule out this possibility in any time before this time window because of the unavailability of medical history. The infection was thought to be acquired after the patient’s abdominal operation, supporting the notion that the gastrointestinal tract is a prominent reservoir for *Candida* colonization [25] and that IAC is an important source of resistant infections [26]. This study underscores the need to be alert about potential echinocandin resistant *C. glabrata* infections even in the absence of typical risk factors [27].

In summary, our results demonstrate that the high-level of echinocandin resistance in the clinical L74 isolate of *C. glabrata* was due to the combination of null function of Fks1 and the novel non-HS point mutation E655 K in Fks2.

## Supplementary Material

Supplemental Material
