# Supplementary material for: Novel FKS1 and FKS2 modifications in a high-level echinocandin resistant clinical isolate of Candida glabrata
Source: Emerg Microbes Infect. 2019 Nov 12;8(1):1619–25. doi: 10.1080/22221751.2019.1684209 (PMC6853239; doi:10.1080/22221751.2019.1684209)
Supplement: Supplemental Material [file TEMI_A_1684209_SM8179.docx]

**Table S1. Sequences of primers and crRNA used in this study**

| **Primer name** | **Sequence (5’-3’)** | **Description** |
| --- | --- | --- |
| *FKS1*-amp-F | ATGTCTTACAATAATAACGGAC | Used for *FKS1* amplification |
| *FKS1*-amp-R | TTATTTGATTGTAGACCAGGTC |  |
| *FKS1*-seq-F | ATGTCTTACAATAATAACGGAC | Used for *FKS1* sequencing |
| *FKS1*-seq-R361-380 | ATAGCGATGGCATTAGGATC |  |
| *FKS1*-seq-F661-680 | GAAGTCGGTTTCAGAAACAT |  |
| *FKS1*-seq-F1381-1400 | TACACACACAACTATCAACA |  |
| *FKS1*-seq-F2161-2180 | TTGGCTACTACTGATATGGA |  |
| *FKS1*-seq-F3001-3020 | GAACTGGAAAACGCTGAATT |  |
| *FKS1*-seq-F3761-3780 | GTGAACAAATGTTGTCCCGT |  |
| *FKS1*-seq-F4711-4730 | GCCCAAACAGGTGTTAAGAC |  |
| *FKS2*-amp-F | ATGTCTTACGATCAAGGTGG | Used for *FKS2* amplification |
| *FKS2*-amp-R | TTATTTTATAGTGGACCAGGTCTT |  |
| *FKS2*-seq-F | ATGTCTTACGATCAAGGTGG | Used for *FKS2* sequencing |
| *FKS2*-seq-R401-420 | ACCGTTGCTGCCCATCCCAT |  |
| *FKS2*-seq-F731-750 | GGTATTTCGCAGCTCAGCTT |  |
| *FKS2*-seq-F1503-1522 | ACTGGTAAACAATCAACCTG |  |
| *FKS2*-seq-F2361-2380 | TGCTATCGACCATGTTCAGA |  |
| *FKS2*-seq-F3161-3180 | TGGATGAAGAACCTCCTTTG |  |
| *FKS2*-seq-F3865-3884 | GAACAAATGTTGTCCCGTGA |  |
| *FKS2*-seq-F4777-4796 | GCAGGTAGTTGTTTCATTGG |  |
| *FKS2*-CRISPR 1 | GGCCACTGTTTTATTCTTCTCG | *FKS2* E655K outside forward primer |
| *FKS2*-CRISPR 2 | CAACTACTACTATGAGATGTACTGG | PAM site mutation forward primer |
| *FKS2*-CRISPR 3 | CCAGTACATCTCATAGTAGTAGTTG | PAM site mutation reverse primer |
| *FKS2*-CRISPR 4 | CCAAGGAGTTAAGATGGAAATACC | *FKS2* E655K outside reverse primer |
| crRNA | ATACTCACCAGTACATCTCAGTTTTAGAGCTATGCT | CRISPR crRNA |
| qPCR CglFKS1-F | CCTCACTATGCTGAAAGAATT | Used for quantification real-time PCR |
| qPCR CglFKS1-R | AGGATCTTGATCATCCATACC |  |
| qPCR CglFKS2-F | ATATGTCTGGTTCGTCAACTCC |  |
| qPCR CglFKS2-R | AACGGGACTTTGTGGATCAG |  |
| qPCR CglFKS3-F | GCGCCAAAGAGAACAAGAAT |  |
| qPCR CglFKS3-R | TGCAACCTGCCTAACCATTT |  |
| CglRDN5.8 F | CTTGGTTCTCGCATCGATGA |  |
| CglRDN5.8 R | GGCGCAATGTGCGTTCA |  |
